# An Evidence-Based, Nursing Handover Standard for a Multisite Public Hospital in Switzerland: Web-Based, Modified Delphi Study

**DOI:** 10.2196/17876

**Published:** 2020-06-15

**Authors:** Nadine Tacchini-Jacquier, Hélène Hertzog, Kilian Ambord, Peter Urben, Pierre Turini, Henk Verloo

**Affiliations:** 1 Valais Hospital Sion Switzerland; 2 Haute École Spécialisée Suisse orientale HES-SO Valais / Wallis Sion Switzerland; 3 Service of Old Age Psychiatry University Hospital of Lausanne Prilly Switzerland

**Keywords:** Delphi survey, consensus, nursing, shift, nursing handover, standard, patient transfers, peripheral hospital

## Abstract

**Background:**

Ineffective communication procedures create openings for errors when health care professionals fail to transfer complete, consistent information. Deficient or absent clinical handovers, or failures to transfer information, responsibility, and accountability, can have severe consequences for hospitalized patients. Clinical handovers are practiced every day, in many ways, in all institutional health care settings.

**Objective:**

This study aimed to design an evidence-based, nursing handover standard for inpatients for use at shift changes or internal transfers between hospital wards.

**Methods:**

We carried out a modified, multiround, web-based, Delphi data collection survey of an anonymized panel sample of 264 nurse experts working at a multisite public hospital in Switzerland. Each survey round was built on responses from the previous one. The surveys ended with a focus group discussion consisting of a randomly selected panel of participants to explain why items for the evidence-based clinical nursing handover standard were selected or not selected. Items had to achieve a consensus of ≥70% for selection and inclusion.

**Results:**

The study presents the items selected by consensus for an evidence-based nursing handover standard for inpatients for use at shift changes or internal transfers. It also presents the reasons why survey items were or were not included.

**Conclusions:**

This modified Delphi survey method enabled us to develop a consensus- and evidence-based nursing handover standard now being trialed at shift changes and the internal transfers of inpatients at our multisite public hospital in Switzerland.

## Introduction

Health care is complex. The processes necessary for communicating health care information are a continuous challenge for health care professionals and health care institutions. Deficient communication processes create the potential for errors when caregivers fail to transfer complete, consistent information [1]. Clinical handovers are practiced every day, in many ways, in all institutional health care settings [2,3]. The clinical handover of patients, according to the Australian Council for Safety and Quality in Health Care’s 2005 clinical handover report [4], concerns and is defined as follows: *the transfer of professional responsibility and accountability for some or all aspects of care for a patient, or group of patients, to another person or professional group on a temporary or permanent basis*.

The literature distinguishes three basic types of good practice in nursing handover: bedside, verbal, and nonverbal. Handovers at the bedside promote face-to-face interaction between patients and nurses and encourage patients to participate verbally, thus putting them at the center of the information exchange process [5,6]. Verbal communication usually takes place in an office setting, where the nurse responsible for a group of patients describes relevant documented information and perhaps gives their professional opinion. Nonverbal communication also usually takes place in an office setting, where nurses inform themselves by reading patients’ health records, including progress notes, medication charts, observation charts, and nursing care plans. Finally, recorded communication can also be used in an office setting if the nurse in charge makes a recording describing the relevant information so that the oncoming shift can listen to it at a convenient time.

Deficient or absent clinical handovers, or failures to transfer information, responsibility, and accountability, can have severe consequences for hospitalized patients [7]. They have been shown to result in delays to diagnosis, treatment, and care; tests being missed or duplicated; and subsequent incorrect operationalization of care plans or drug follow-up [8].

More than a decade ago, the World Health Organization (WHO) collaborating centers on patient safety strongly recommended that their members improve communication during patient handovers by declaring the following: “Ensure that health-care organizations implement a standardized approach to handover communication between staff, change of shift and between different patient care units in the course of a patient transfer” [9]. Unfortunately, the world’s countries were not universally proactive in addressing this recommendation and implementing structured, evidence-based handovers to improve patient safety and the continuity of care. Countries such as Australia, Belgium, China, Spain, the United Kingdom of Great Britain and Northern Ireland, the United States, and the Netherlands developed national and regional standards for nursing and interprofessional handovers [4,10-16]. Despite a recent systematic review by Bukoh and Siah, which demonstrated that structured handovers reduced incidences of patient complications, medication errors, and general adverse events, none of the handover standards examined had been designed using a robust evidence-based methodology. As a consequence of this lack of strong evidence, countries and health care institutions naturally hesitate to adopt standardized handovers. To the best of our knowledge, there are currently no national, regional, or local evidenced-based nursing handover standards in use in Switzerland [17]. Although some Swiss university hospitals are working hard to implement more structured nursing handover systems, no national policy is available as yet. This research is a first step toward the development of a more widespread nursing standard of evidence-based handover communication. It will support the nursing experts who are declaring that patient safety will be improved by the implementation of care delivery systems that effectively structure handover communication [8].

Nursing handover practices in our multisite hospital in Switzerland were highly variable, sometimes unreliable, and differed across medical specialties. This led to inconsistencies in the content and accuracy of handover information. Preceding studies have revealed multiple barriers to communication within health care organizations, including hierarchy, gender, ethnic background, primary health care education, and different communication styles [18,19]. Inconsistencies in communication may cause substantial risks to patient safety and care [20]. Other health care institutions have recently tried to uncover the specific risks and contributing factors to difficulties in handover communications [21]. In 2017, an internal survey of health care professionals at a multisite public hospital in Switzerland, concerning its culture of patient care safety, revealed that almost two-thirds of them (ie, nurses, physicians, and allied health care professionals) considered the quality of information transmission to be deficient and a risk to the safety of their patients [22]. Intervention studies have shown that information is poorly preserved if verbal or handwritten handovers are transferred across multiple shifts [23], rather like a game of broken telephone.

Validated causes at the root of handover communication failures include institutional cultures that fail to promote effective handovers (eg, lack of teamwork and respect); the different expectations of information givers and receivers; inadequate methods of communication, whether verbal, recorded, bedside, or written; ill-timed or badly coordinated physical transfers and patient handovers; interruptions to, or the lack of time allocated to, successful handovers; nonstandardized handover procedures; insufficient staff to ensure effective handovers at pertinent times of the day or week; and a lack of participation by patients during their handovers [24-26].

The web-based, modified, electronic Delphi (e-Delphi) survey presented here developed standardized solutions to these risks and then developed and implemented factors to improve the effectiveness of communication during transitions of care [27]. It has been proposed that efforts to standardize the content and processes of patient handovers (eg, shift reports) ensure consistency in the exchange of vital information and effectively improve communication and, thus, patient safety [28,29]. Despite few details about what the precise contents of any handover communication should be, standardizing processes (eg, describing the patient) could be a starting point for choosing the contents (eg, patient name, age, and current condition). To ensure that information transfer in complex care environments is safe and effective, specific information about each process should form a part of any two-way communication [30]. There is little empirical evidence in the current literature of any link between patient safety and the effective transfer of information during handovers [31].

We used the Delphi survey method as our framework for a handover content–selection process based on the results of several rounds of questionnaires sent to a selected panel of nurse experts [32]. This approach, according to Tong et al [33] and the World Medical Association [34], used the following: *structured anonymous communication between experts to gather consensus perspectives about an issue or topics that can then be used to inform decision making or to agree about methods of functioning*.

An e-Delphi study involves a number of rounds of web-based questionnaires in which experts are requested to provide their opinions on precise topics [35]. They do so independently, but after the first round, they are aware of the other participants’ aggregated opinions when making their second-round decisions. The goal is to reach a consensus. The e-Delphi method’s key features are iteration and anonymity, which were found to be particularly advantageous for a multisite hospital dealing with several medical specialties. The anonymous, web-based format encourages participation and honest opinion sharing by large numbers of panel members and prevents senior or influential individuals from monopolizing or influencing discussions. This is important in the hierarchical environment of a health care institution.

The higher the number of handovers, the more significant risks patients face, although little is known about the exact mechanisms by which handovers destabilize care. Information management at nursing shift changes has been highlighted as being particularly prone to mistakes [23,31]. The general themes involved in clinical nursing handover standards are affected by a range of factors that combine to define how smooth and safe they are for patients [26,36]. A nursing handover is a vital element in the continuity of care [37]. Transitions in care are notable periods of vulnerability in a patients’ treatment journey [38]. Transferring responsibility for a patient’s care to another health care professional increases the chances of an error occurring, especially if key information is communicated inaccurately and inefficiently [39]. Any inaccurate, unclear, or incomplete transfer of information increases the risks of potentially severe errors [40,41].

This study aimed to use a modified e-Delphi survey to design an evidence-based, nursing handover standard for inpatients for use at shift changes or internal transfers between the hospital wards of a multisite public hospital in Switzerland.

## Methods

### Design

#### Overview

Study design was based on a previously published protocol [42] describing the use of a multiround survey of a targeted panel sample of 264,300 nurse experts to build a consensus for the contents of an evidence-based nursing handover standard. A rounds-step Delphi technique documented by Keeney and al [30], Burchell and al [43], Slade and al [44], and Cole et al [31] was used for this study. Formal reporting on the qualitative data from responses to open questions and on the focus group was based on a checklist of the most common methods of data collection in qualitative health care research [33].

#### Comprehensive Scoping Review to Design the Components of a Web-Based Modified E-Delphi Survey

A comprehensive scoping review of the literature was made to find the components of effective, evidence-based, clinical nursing handovers. Predefined terms were used to search for published articles in the following electronic databases, from inception until September 30, 2018: MEDLINE (Medical Literature Analysis and Retrieval System Online) via PubMed (from 1946), Embase (from 1947), CINAHL (Cumulative Index to Nursing and Allied Health Literature) (from 1937), Web of Science (from 1900), ScienceDirect, and Wiley. The bibliographies of all relevant articles were hand-searched, and Google Scholar was used to search for unpublished studies.

### Data Collection Process

Data collection was preceded by a comprehensive, systematic scoping review of the components of an evidence-based clinical nursing handover standard. This enabled us to draw up a list of potential handover items to be decided on using a web-based, modified e-Delphi survey. Data collection began in mid-September 2018 and ended in mid-December 2018.

### Setting and Population

The study was conducted at a multisite public hospital that recorded over 40,000 individual hospitalizations in 2018; it is composed of two hospital centers in two linguistically and culturally different regions of a single Swiss canton [20]. Each hospital center has standard medical hospitalization wards to fulfil its mission of providing general public health care; however, the more complex medical specialties are only present at the French-speaking hospital center. The French-speaking hospital center has 39 acute care and eight psychiatric wards, with 1134 full-time-equivalent health care professionals. The German-speaking hospital center has 15 acute care and three psychiatric wards, with 390 full-time-equivalent health care professionals. Each acute care ward has a triad of nurse experts: a registered nurse clinical-educator, a student-success coach, and a nurse supervisor. Each hospital center’s nursing managers and departmental supervisors supported and encouraged eligible staff to participate actively in the data collection process. We aimed for a targeted anonymized panel of nurse experts from both the French-speaking and German-speaking hospital centers.

### Knowledge Synthesis for the Selection of Items for the Nursing Handover Standard

Investigators examined the review’s findings at two item-selection meetings and chose the potentially relevant components of an evidence-based nursing handover standard to be included in the e-Delphi panel survey. Textbox 1 presents the main potential components. The questionnaire was made available in French and German; it was trialed with four clinical experts not involved in the study, who were asked to assess items for clarity, wording, and understandability.

**Relevant components of an evidence-based nursing handover standard for inclusion in the electronic Delphi (e-Delphi) survey.**

*Culture and attitude for good handover practices:*

- Respectful and collaborative attitude
- Proactive listening
- Positive, factual language adapted to patients, situations, and professionals
- Confidentiality
- The handover environment

*Handover preparation, including coordination and sources of information:*

- Clinical assessment before the handover
- Use different sources of information
- Updated patient records
- Reconsider and reanalyze information

*Handover phases, including communication of patient-specific information:*

- Mnemonic techniques to guide communication and format content chronologically
- Face-to-face handovers with the opportunity to ask questions
- Information technology to support data access to the patient’s complete history and health status
- Patient records ensuring the traceability of decisions and follow-up
- Information technology to support data updates
- Flexible information technology to support adaptations for each specialized ward
- Handovers at the patient’s bedside at the risk of reduced confidentiality
- Handovers at the patient’s bedside for understanding their values and preferences

*A minimum dataset should be transmitted:*

- Summary of the patient’s hospitalization history and care planning
- Assessment of the disease
- Prognosis of health status
- Allergies
- Reanimation status
- Medication treatment
- Laboratory results
- Vital signs
- Patient’s activities and planned examinations

### Eligibility of Nurse Experts

In collaboration with each center’s director of nursing, investigators invited eligible, professionally active nurse experts from the general medicine, surgery, geriatrics and rehabilitation, intensive care, emergency, maternity and gynecology, and psychiatric wards to join our panel and express their opinions.

The eligible sample population was composed of 264 nurse experts. They were all highly qualified, very experienced, and recognized as such within their departments.

**Figure 1:**
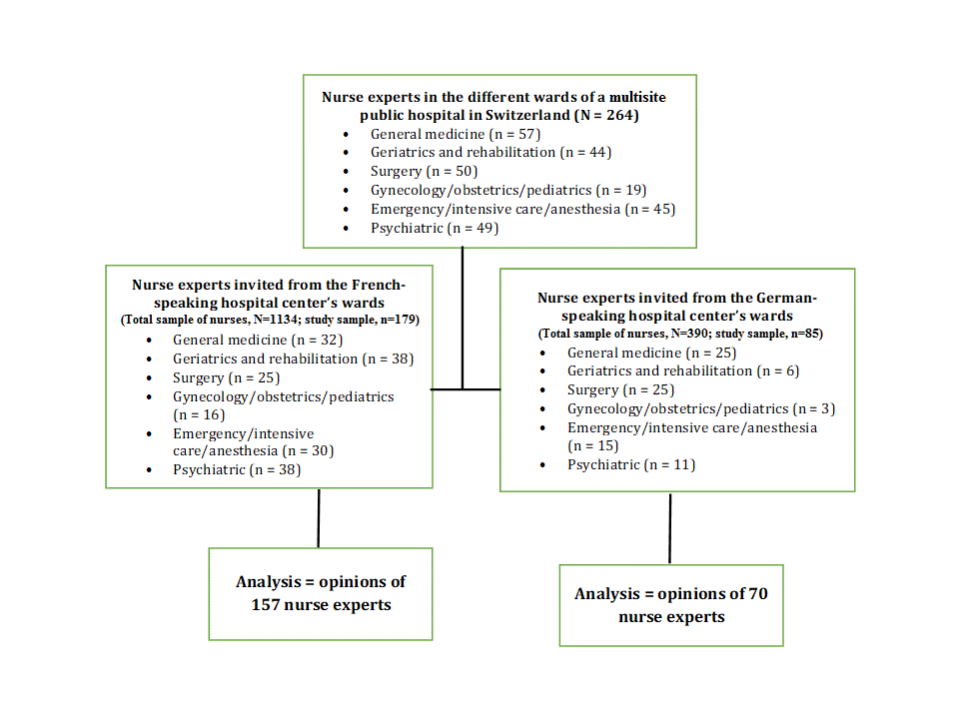
Selection of panels of all nurse experts from the hospital centers in the French- and German-speaking regions.

Inclusion criteria were as follows: (1) to have worked in their current specialty for at least three months before the start of the data collection process; (2) to have been employed as a registered nurse clinical-educator, student-success coach, or nurse supervisor with recognized knowledge and expertise in their field; and (3) to have the willingness and time to participate and the capacity to understand and give an opinion on clinical statements. In agreement with the two hospital centers’ directors, all eligible nurse experts were invited to participate in the e-Delphi survey. Figure 1 presents the recruitment process.

### Survey Administration

The Human Research Ethics Committee of the Canton Vaud (CER-VD) (2019-00925) approved the study, participants’ anonymity was ensured, and the standards of good research practice mentioned in the Declaration of Helsinki were respected [34]. The directors of both hospital centers approved the study [45]. Each round of the e-Delphi survey was transmitted using SurveyMonkey, a secure, commercial, web-based platform that ensures anonymous survey participation. Data were stored in Switzerland, protected using high-security firewalls, and treated confidentially. All eligible nurse experts were sent a personalized link to each round of the survey. Although a personalized link was used to access the survey, and sociodemographic and professional characteristics were stored, names and contact details were removed from the completed survey. Survey items had to reach a predetermined 70% rate of consensus for inclusion in the standard [30].

### The E-Delphi Process

**Figure 2:**
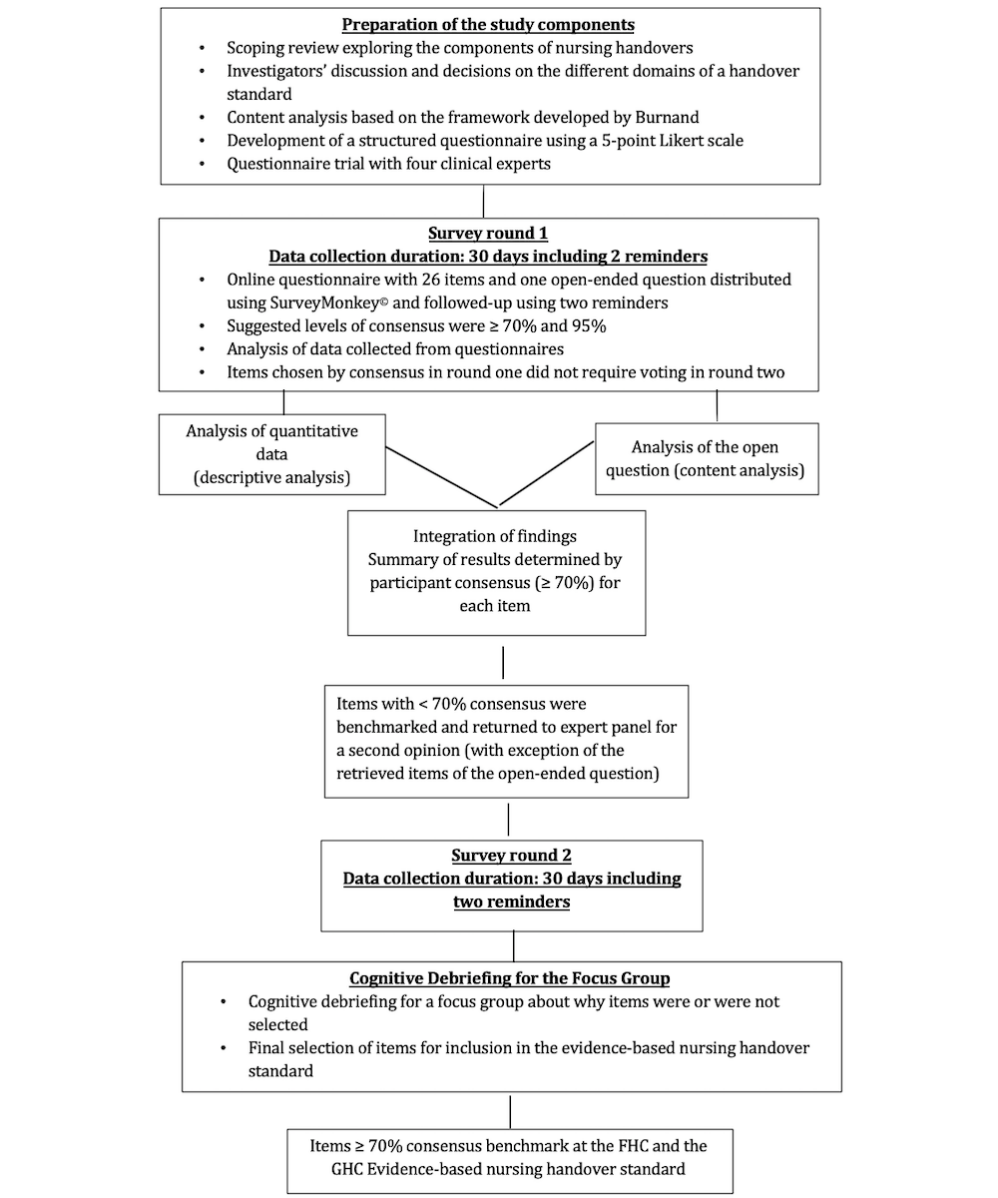
E-Delphi survey data collection process for designing an evidence-based nursing handover standard. FHC: French-speaking hospital center; GHC: German-speaking hospital center.

The modified e-Delphi data collection process was composed of three rounds. In round 1, potential nurse-expert panelists received an email asking them to give their opinions on 26 items in a structured questionnaire (see Figure 2). A cover letter described the study’s aims, gave instructions on how to fill in the questionnaire, and provided assurances that participants’ anonymity would be guaranteed. Filling in the online questionnaire was considered as a proxy for the nurse experts giving their written informed consent to participate. The survey used a 5-point Likert scale, ranging from *Strongly agree* (scoring 5) to *Strongly disagree* (scoring 1), to describe participants’ opinions on whether items should be included in the evidence-based clinical nursing handover standard. A final, open-ended question asked, “What topic, not yet mentioned in these statements, should also be integrated into the handover standard?”

Respondents explained their choices or suggested items not listed in the first round, but which they believed were important. Two email reminders were sent out to nonresponders 1 and 2 weeks after launching the e-Delphi process. Round 1 closed after 30 days, and all the returned data were analyzed.

Round 2 was transmitted along with a second instructional cover letter asking participants to give their opinions on the 11 new items suggested by their peers via round 1’s open question. Two email reminders were sent out to nonresponders 1 and 2 weeks after the start of round 2. Round 2 closed after 30 days, and all the returned data were analyzed.

### Cognitive Debriefing of the Focus Group

The study’s third part was a cognitive debriefing. Patrick et al outlined how a cognitive debriefing process is structured around, and usually focused upon, the assessment of a specific clinical output; it should incorporate direct questions about participants’ understandings of the measures leading to that output, as well as their relevance and comprehensiveness [46]. The cognitive debriefing’s primary aim was to collect data from a focus group of volunteer participants who discussed and explained the findings and validated the consensus items to be used in the handover standard. A secondary aim was to better understand why some items had not reached the required level of consensus and to explore influencing factors.

Focus group participants were selected using a purposive sampling strategy aiming to represent different nursing roles, at different hierarchical levels, and in different languages at our multisite public hospital. They included registered nurses, nurse supervisors, registered nurse clinical-educators, student-success coaches, the directors of nursing from the French-speaking and German-speaking hospital centers, the nursing quality and risk manager, the nurse manager for electronic patient records, and lecturers in nursing sciences from the University of Applied Sciences in Nursing as facilitators. All the participants were directly involved in the implementation of evidence-based nursing handover standards in their respective environments. Cognitive debriefings have been documented as good research practice for gaining a better understanding of participants’ agreements and disagreements about survey item statements [47].

The cognitive debriefing took place in December 2018 in an appropriate seminar room of our multisite public hospital’s central administrative area. The room was large enough to enable all the participants to sit in a circle, ensuring visibility for everyone. All the participants had received prior verbal and written information about the session’s aims, the data collection procedure, the focus group’s principles, and the use that would be made of the data. Participants gave their written informed consent for the cognitive debriefing to be audio recorded for transcription. Participants received the results for the items voted on in the two-round survey. The cognitive debriefing was conducted by a moderator presenting item by item, accompanied by an observer, who began with the question, “Could you explain or hypothesize why a consensus was reached on some items but not others?”; this was used as a reminder throughout the debriefing to keep the participants focused. Figure 2 gives a schematic representation of the e-Delphi data collection process.

### Data Analysis

The sociodemographic characteristics of the entire nurse-expert panel were also retrieved using SurveyMonkey, including age and years of experience in their professional role. All the items were available in French and German. Data were extracted onto a Microsoft Excel spreadsheet and subsequently imported into SPSS, version 25.0, statistical software (IBM Corp) [48].

The data collection process involved three rounds. Round 1 closed after 30 days, and the collected data were analyzed. Each item was described using descriptive statistics, such as frequency, distribution, mean (SD), and median (IQR-75). An appropriate exact test was used to compare means and percentages. A consensus agreement was defined using dichotomized *yes*/*no* answers for each item’s statement, with *Strongly disagree*, *Partially disagree*, and *No opinion* recoded as *no* answers for that item, and *Partially agree* and *Strongly agree* recoded as *yes* answers. Round 2 was composed of the statements for which no consensus had been reached in round 1, plus additional statements that had arisen from the panel’s suggestions in response to the open-ended question. Round 2 ended after a further 30 days, and the returned data were analyzed as mentioned previously.

The level of consensus chosen for accepting an item was set at ≥70% of *yes* answers. Questionnaires returned with more than 20% of their items unanswered were excluded from the analysis.

The third and final round involved the cognitive debriefing of a focus group made up of 15 randomly selected but highly motivated nursing experts. Qualitative data collected during the focus group were transcribed and analyzed using deductive thematic content analysis [49,50] in NVivo 12 software (QSR International) [51]. Transcripts were read, themes and subthemes emerging from the data were coded, and an analysis map was drawn to manage them. The coauthors approved this analytical process, and disagreements were resolved via discussion.

## Results

### Scoping Review

The systematic scoping review of the literature enabled the investigators to prepare 22 item statements that were classified into three domains of an evidence-based nursing handover standard: the handover environment, the handover preparation phase, and the handover phase itself. The overall handover process should have a structure, defined content for information or communication, be supported by information technology (IT) and electronic patient records, specify the type of handover, and include any pertinent education or training information (not treated in this study). Four extra items, not drawn from the scoping review, were integrated into the questionnaire; these related to the principles of collaborative practice considered in the charter of good practices in interprofessional health care collaboration, as edited by the Swiss Academy of Medical Sciences [52].

### Response Rate

From the maximum potential eligible sample (N=264) of invited nurse experts, 245 returned their round 1 questionnaires (an excellent response rate of 92.8%), and 227 met the requirements for analysis (valid response rate of 86.0%). The round 1 response rates for the French-speaking and German-speaking hospital centers were 87.7% (157/179) and 82% (70/85), respectively. In round 2, 201 participants completed the study and met its requirements (valid response rate of 76.1%), with the response rates for the French-speaking and German-speaking hospital centers being 75.4% (135/179) and 78% (66/85), respectively.

### Nurse Experts’ Sociodemographic and Professional Characteristics

Most nurse experts were female (176/216, 81.5%), trained in clinical nursing, and working as registered nurse clinical-educators, student-success coaches, and nurse supervisors. The average respondent was 41.0 years old (SD 9.6) with a mean of almost 18 years of professional experience (SD 9.5). Two-thirds of invited nurse experts were working in the surgery and general medicine wards (see Table 1).

**Table 1:** Participants’ sociodemographic and professional characteristics.

Sociodemographic and professional characteristics			French-speaking region’s hospital center (n=157)		German-speaking region’s hospital center (n=70)		Multisite public hospital in Switzerland (N=227)
**Sex^a^, n (%)**							
	Men	32 (21.5)		8 (12)		40 (18.5)	
	Women	117 (78.5)		59 (88)		176 (81.5)	
**Age (years)**							
	Mean (SD)	42.0 (9.6)		38.9 (9.2)		41.0 (9.6)	
	Median	42		40		41	
	Min-max	27-60		26-61		26-61	
**Nurse expert’s profession^b^, n (%)**							
	Student-success coach	43 (30.5)		13 (19)		56 (26.9)	
	Registered nurse clinical-educator	47 (33.3)		25 (37)		72 (34.6)	
	Nurse supervisor	33 (23.4)		19 (28)		52 (25.0)	
	Other^c^	18 (12.8)		10 (15)		28 (13.5)	
**Years of experience**							
	Mean (SD)	18.4 (9.2)		16.2 (9.9)		17.7 (9.5)	
	Median	17.5		15		17	
	Min-max	4-42		4-40		4-42	

^a^Number of respondents for this question was n=149 (French), n=67 (German), and n=216 (total).

^b^Number of respondents for this question was n=141 (French), n=67 (German), and n=208 (total).

^c^Professionals holding the official role of ward expert and educated to the level of Registered Nurse or Bachelor of Nursing Science.

### First-Round Participants and Findings

#### Participants

Participants were registered nurse clinical-educators (72/208, 34.6%), student-success coaches (56/208, 26.9%), and nurse supervisors (52/208, 25.0%); 13.5% (28/208) were registered nurses or held a Bachelor of Nursing Science degree without postgraduate training. Table 1 presents the characteristics of participants in the French-speaking and German-speaking hospital centers.

#### Findings

Nurse experts had a high rate of agreement with regard to most of the items. However, the French-speaking hospital center’s nurse experts did not reach a round 1 consensus of ≥70% on the following items: *Information technology support flexibility allows adaptability for each specialized unit*, *Handovers at the patient’s bedside enable a better understanding of patient values and preferences*, *Handovers at the patient’s bedside risk compromising confidentiality of patient health and nursing data*, *Provide a list of medication*, and *Present laboratory results*.

The German-speaking hospital center’s nurse experts failed to find a round 1 consensus of ≥70% on two items, namely, *A mnemonic technique guiding patient transfer in a chronological way* and *Present laboratory results*.

**Figure 3:**
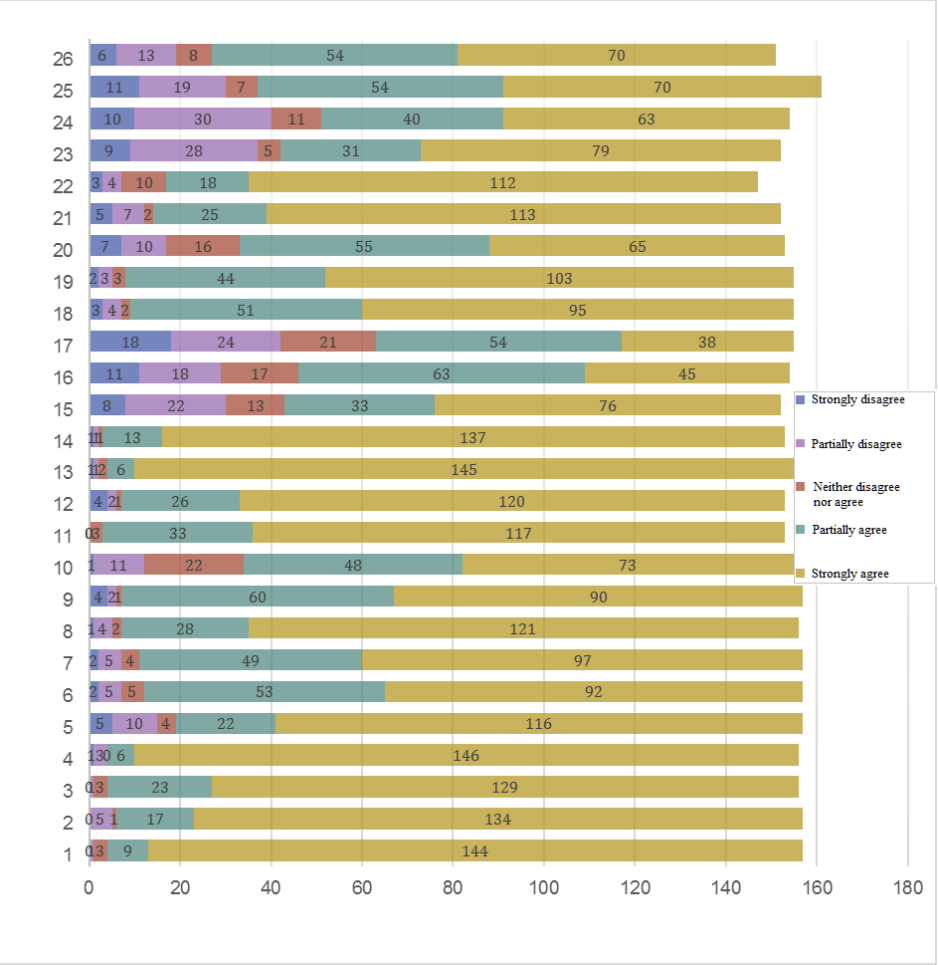
Distribution of round 1 opinions on nursing handover items given by the panel of nurse experts from the French-speaking region’s hospital center (n=179). The numbers of participants who rated each item according to the legend options are indicated within the respective colored portions of each bar. The 26 items, within their respective categories, are listed here. Good handover practices are carried out in a collaborative spirit: 1. Adopt a respectful and collaborative attitude; 2. Adopt proactive listening; 3. Use positive, factual language adapted to patients, situations, and professionals; 4. Respect confidentiality; and 5. Conduct the handover in a calm and quiet environment to prevent interruptions. The preparatory phase for handover includes the coordination of activities to gather the different sources of information to be communicated: 6. Make a clinical assessment before the handover; 7. Regroup different sources of information; 8. Update patient records; and 9. Reconsider and reanalyze information. The handover phase itself should include the communication of all patient-specific information: 10. Use a mnemonic technique to guide communication and format content chronologically; 11. Face-to-face handovers give nurses the opportunity to ask questions; 12. Information technology (IT) should support data access to patient’s complete history and health status; 13. Patient records should allow the traceability of decisions and follow-up; 14. IT should support data updates; 15. Flexible IT support should allow for adaptability for each specialized unit; 16. Handovers at the patient’s bedside risk breaching confidentiality; and 17. Handovers at the patient’s bedside enable a better understanding of their values and preferences. A minimum dataset should be transmitted: 18. Provide a summary of patient’s hospitalization history and care planning; 19. Provide an assessment of the disease, including severity; 20. Present a prognosis of health status; 21. Provide a list of allergies; 22. Present a reanimation status; 23. Provide a list of medication; 24. Present laboratory results; 25. Update vital signs; and 26. Provide a list of all patient activities.

**Figure 4:**
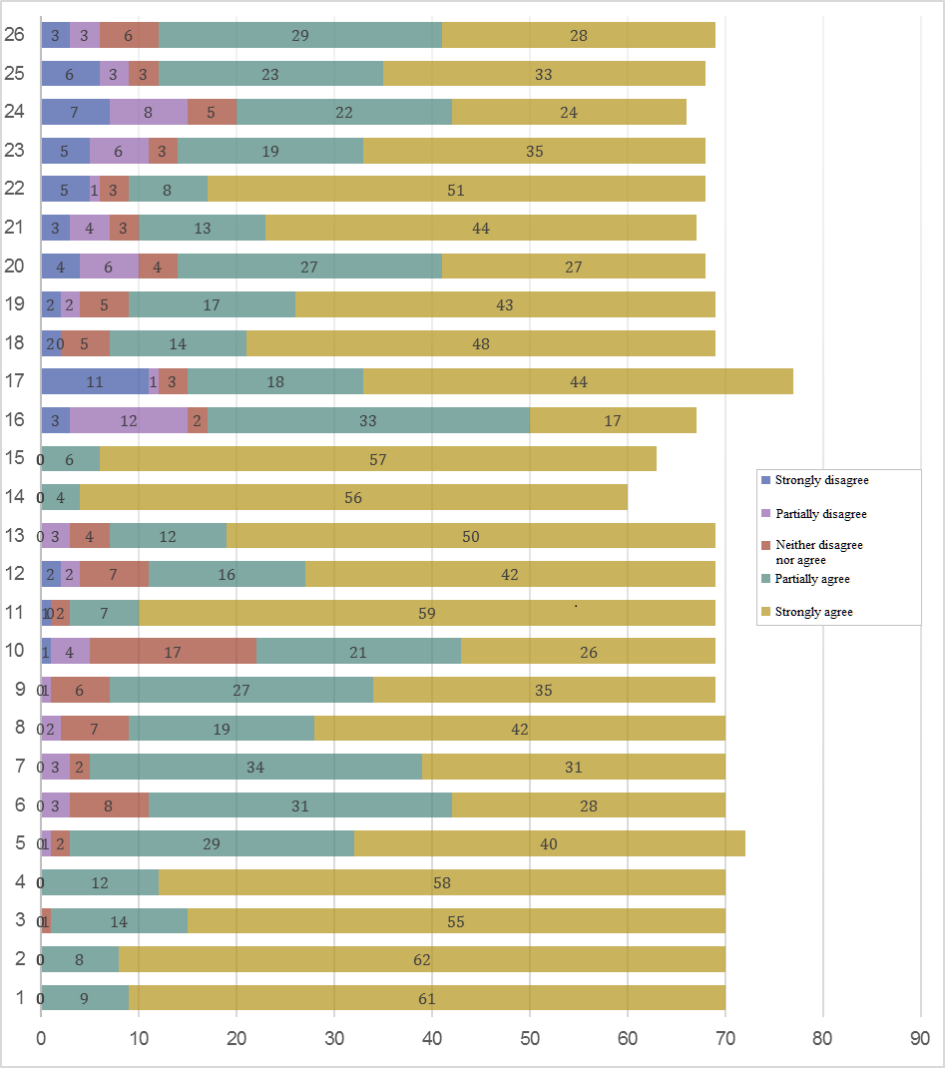
Distribution of round 1 opinions on nursing handover items given by the panel of nurse experts from the German-speaking region’s hospital center (n=85). The numbers of participants who rated each item according to the legend options are indicated within the respective colored portions of each bar. The 26 items, within their respective categories, are listed here. Good handover practices are carried out in a collaborative spirit: 1. Adopt a respectful and collaborative attitude; 2. Adopt proactive listening; 3. Use positive, factual language adapted to patients, situations, and professionals; 4. Respect confidentiality; and 5. Conduct the handover in a calm and quiet environment to prevent interruptions. The preparatory phase for handover includes the coordination of activities to gather the different sources of information to be communicated: 6. Make a clinical assessment before the handover; 7. Regroup different sources of information; 8. Update patient records; and 9. Reconsider and reanalyze information. The handover phase itself should include the communication of all patient-specific information: 10. Use a mnemonic technique to guide communication and format content chronologically; 11. Face-to-face handovers give nurses the opportunity to ask questions; 12. Information technology (IT) should support data access to patient’s complete history and health status; 13. Patient records should allow the traceability of decisions and follow-up; 14. IT should support data updates; 15. Flexible IT support should allow for adaptability for each specialized unit; 16. Handovers at the patient’s bedside risk breaching confidentiality; and 17. Handovers at the patient’s bedside enable a better understanding of their values and preferences. A minimum dataset should be transmitted: 18. Provide a summary of patient’s hospitalization history and care planning; 19. Provide an assessment of the disease, including severity; 20. Present a prognosis of health status; 21. Provide a list of allergies; 22. Present a reanimation status; 23. Provide a list of medication; 24. Present laboratory results; 25. Update vital signs; and 26. Provide a list of all patient activities.

Figures 3 and 4 present the participant ratings for the 26 statements by French-speaking and German-speaking hospital centers, respectively.

Table 2 presents the mean item scores and distributions of consensus agreement on the item statements submitted to the panel of nurse experts with the lead question, “To what extent do you agree that the following items should be an integral part of handovers?” Consensus required ≥70% agreement with the statement, taken as the sum of the *yes* Likert-scale options *Strongly agree* and *Partially agree*. The mean (SD) and median (IQR-75) was calculated for each item.

The open question enabled each respondent to propose supplementary items, not mentioned in round 1, for integration and submission to the panel of nurse experts in round 2. In round 2, the German-speaking hospital center failed to submit new topics proposed by the French-speaking hospital center to its staff, resulting in some heterogeneity in the choice of round 2 items (see Table 3).

Respondents from both the French-speaking and German-speaking hospital centers proposed second-round items to do with the patient’s identity, their social context (eg, living alone or with relatives), their expectations and those of their families or relatives, and discharge planning. Additionally, respondents from the French-speaking hospital center suggested the following round 2 items: (1) handover duration should be chosen by the wards involved, (2) time of day should be chosen by the wards involved, (3) conditions of hospitalization (eg, elective or emergency and whether the patient was sectioned), (4) advanced health care directives, and (5) any identified clinical risks during hospitalization. Respondents from the German-speaking hospital center proposed adding a second-round item on the risks of transmitting infection.

**Table 2:** Analysis of the survey statement scores from the French-speaking and German-speaking hospital centers.

Statements and their categories		French-speaking hospital center (n=157)			German-speaking hospital center (n=70)			
		Mean (SD)^a^	Median (IQR-75)	Consensus, %		Mean (SD)	Median (IQR-75)	Consensus, %
**Good handovers are carried out in a spirit of cooperation**								
	1. Adopt a respectful and cooperative attitude	4.9 (0.4)	5 (5)	97.5		4.8 (0.3)	5 (5)	100
	2. Adopt proactive listening	4.8 (0.6)	5 (5)	96.2		4.9 (0.3)	5 (5)	100
	3. Use positive, factual language adapted to patients, situations, and professionals	4.8 (0.6)	5 (5)	96.8		4.7 (0.4)	5 (5)	98.6
	4. Respect confidentiality	4.9 (0.6)	5 (5)	96.8		4.8 (0.4)	5 (5)	100
	5. Conduct the handover in a calm, quiet environment to prevent interruptions	4.5 (1.0)	5 (4)	87.9		4.5 (0.5)	5 (5)	98.6
**The preparatory phase for handover includes the coordination of activities to gather the different sources of the information to be communicated**								
	6. Make a clinical assessment before handover	4.4 (0.8)	5 (4)	92.4		4.0 (0.8)	4 (4)	84.3
	7. Gather different sources of information	4.5 (0.8)	5 (5)	93.0		4.3 (0.7)	4 (4)	92.9
	8. Update patient records	4.6 (0.7)	5 (5)	95.0		4.4 (0.8)	5 (4)	87.1
	9. Reconsider and reanalyze information	4.4 (0.7)	5 (4)	95.5		4.4 (0.8)	5 (4)	88.6
**The information transmission phase should include the communication of all patient-specific information**								
	10. Use a mnemonic technique to guide communication and format content chronologically	4.4 (1.1)	5 (4)	77.1		4.0 (1.1)	4 (4)	67.1^b^
	11. Use face-to-face handovers, which give nurses the opportunity to ask questions	4.8 (0.8)	5 (5)	95.5		4.8 (0.8)	5 (5)	94.3
	12. Information technology should support access to data on the patient’s complete history and health status	4.7 (1.0)	5 (5)	93.0		4.4 (1.1)	5 (4)	82.9
	13. Patient records should enable the traceability of decisions and follow-up	4.9 (0.6)	5 (5)	96.2		4.6 (0.9)	5 (5)	88.6
	14. Information technology should support data updates	4.9 (0.8)	5 (5)	95.5		5.0 (1.2)	5 (5)	85.7
	15. Flexible information technology support should allow for adaptability by each specialized unit	4.1 (1.5)	5 (4)	69.0^b^		4.9 (1.0)	5 (5)	90.0
	16. Handovers at the patient’s bedside risk breaching confidentiality	3.8 (1.3)	4 (4)	68.8^b^		4.0 (1.5)	4 (4)	71.4
	17. Handovers at the patient’s bedside enable a better understanding of their values and preferences	3.5 (1.4)	4 (4)	58.6^b^		4.7 (1.1)	4 (5)	88.6
**A minimum dataset should be transmitted**								
	18. Provide a summary of the patient’s hospitalization history and care plans	4.5 (0.9)	5 (4)	93.0		4.6 (1.0)	5 (5)	88.6
	19. Provide an assessment of the disease, including severity	4.6 (0.9)	5 (5)	93.6		4.4 (1.1)	5 (4)	85.7
	20. Present a prognosis of health status	4.1 (1.3)	4 (4)	76.4		4.3 (1.4)	5 (4)	77.1
	21. Provide a list of allergies	4.6 (1.2)	5 (5)	87.9		4.5 (1.4)	5 (5)	81.4
	22. Present the patient’s reanimation status	4.8 (1.3)	5 (5)	82.8		4.6 (1.3)	5 (5)	84.3
	23. Provide a list of medication	4.1 (1.6)	5 (4)	69.0^b^		4.2 (1.5)	5 (4)	77.1
	24. Present laboratory results	3.8 (1.5)	4 (4)	65.6^b^		4.0 (1.8)	4 (4)	65.7^b^
	25. Provide an update on vital signs	4.2 (1.5)	4 (4)	73.2		4.2 (1.4)	4.5 (4)	80.0
	26. Provide a list of all patient activities	4.3 (1.4)	4 (4)	79.0		4.1 (1.1)	4 (4)	81.4

^a^The survey used a 5-point Likert scale, ranging from *Strongly agree* (scoring 5) to *Strongly disagree* (scoring 1), to describe participants’ opinions on whether items should be included in the evidence-based clinical nursing handover standard.

^b^Nonconsensus: <70% of the nurse experts accepted the item as a necessary, evidence-based, nursing standard for patient handovers.

**Table 3:** Analysis of scores of survey statements failing to reach consensus and items suggested from the open question from the French-speaking and German-speaking hospital centers.

Items from open question and their categories		French-speaking hospital center (n=135)			German-speaking hospital center (n=66)		
		Mean (SD)^a^	Median (IQR-75)	Consensus, %	Mean (SD)^a^	Median (IQR-75)	Consensus, %
**Resubmitted item^b^**							
	28. Handovers at the patient's bedside ensure continuity, quality, and safety of care	3.4 (1.3)	4 (3)	62.2^c^	4.3 (0.8)	4 (4)	71.4
**A minimum dataset should be transmitted (complement by expert, round 1)**							
	29. Identify the patient	4.8 (0.5)	5 (5)	95.6	4.0 (1.2)	5 (4)	78.8
	30. Present the patient’s social context	4.4 (0.8)	5 (4)	89.6	3.5 (1.2)	4 (4)	71.2
	31. Present the patient’s expectations	4.4 (0.8)	5 (4)	89.6	3.8 (1.2)	4 (4)	73.8
	32. Present the patient’s discharge plan	4.6 (0.6)	5 (5)	94.1	4.4 (0.8)	5 (4)	90.9
	33. Risk of transmitting infections^d^	N/A^e^	N/A	N/A	4.2 (1.2)	4 (4)	83.1
	36. State of hospitalization^f^	4.2 (1.1)	5 (4)	80.7	N/A	N/A	N/A
	37. Advanced care directives^f^	4.4 (1.1)	5 (4)	87.4	N/A	N/A	N/A
	38. Present identified clinical risks^f^	4.6 (0.8)	5 (5)	92.6	N/A	N/A	N/A
**The organization provides the right framework for transfer situations and handovers (complement by expert, round 1)**							
	35. Decide on the time of day for handover to ensure continuity of care^f^	4.5 (0.9)	5 (5)	91.1	N/A	N/A	N/A
	34. Define the time required for handover, depending on the situation^f^	4.1 (0.9)	4 (4)	83.0	N/A	N/A	N/A

^a^The survey used a 5-point Likert scale, ranging from *Strongly agree* (scoring 5) to *Strongly disagree* (scoring 1), to describe participants’ opinions on whether items should be included in the evidence-based clinical nursing handover standard.

>^b^Handovers at the patient's bedside ensure continuity, quality, and safety of care was the only resubmitted item that failed to reach the consensus level of agreement of ≥70% from the French-speaking hospital center’s nurse experts.

^c^Nonconsensus: <70% of the nurse experts accepted the item as a necessary, evidence-based, nursing standard for patient handovers.

^d^For the French-speaking hospital center, the Risks of transmitting infections was integrated into item 38, Present identified clinical risks.

^e^N/A: not applicable.

^f^The German-speaking hospital center’s organizers, investigators, or management failed to transmit the suggestions in round 1’s open question made by the French-speaking hospital center.

### Second-Round Participants and Findings

#### Participants

As already mentioned, not all of the additional topics proposed in answer to the open question were submitted to participants at both hospital centers. Second-round respondents had all participated in the first round, and the second-round response rate was 76.1% of all the originally invited participants (201/264). Second-round sociodemographic and professional characteristics were similar to those of the first round (see Table 1).

#### Findings

Table 3 presents the mean second-round item scores for respondents in the two hospital centers. As already mentioned, round 2’s list was composed of the items that failed to reach the overall consensus level in round 1 as well as items suggested in round 1’s open question (ie, question 27) , but the German-speaking hospital center’s organizers, investigators, or management failed to transmit the suggestions made by the French-speaking hospital center.

### Cognitive Debriefing Using a Focus Group

The cognitive debriefing was done with a focus group composed of a purposive sample of participants chosen to discuss the study’s findings.

#### Participants and Duration

Of 18 nurse experts invited to participate in the focus group, 15 (83%) attended the session, including 4 men (27%) and 11 women (73%). The session, including the introduction and conclusion, lasted 60 minutes.

#### Findings

##### Survey Perceptions

The nurse experts gave the survey an encouraging response overall, expressing positive expectations for the study’s final goal and their willingness to create safe, standardized, evidence-based, communication practices for use during shift-to-shift nursing handovers. The following quote illustrates that positive attitude:

A handover standard would reduce the differences in practice caused by each professional’s level of experience and sensitivities.

The survey’s high response rate was testament to its favorable reception from frontline nursing staff. Its results also gave them a base from which to subsequently adapt the standard by adding the content necessary for nursing shift handovers within specific wards or transfers between particular specialties.

##### The Institution’s Role in the Handover Process

Participants expressed the important role of organizational issues (enough time, suitable staffing levels, appropriate environments, etc) in well-functioning nursing handovers, as shown in the item list. Organizational issues are always present in hospital systems, but health care professionals often perceive them to be obstacles. Designing new nursing handover standards was viewed as an opportunity to align the visions of management and clinicians. Participants also mentioned the limitations and risks related to changes in practice. The following quote illustrates this:

Organizational limitations meant that, in general, professionals had not adopted a consensus on how to carry out handovers at the patient's bedside.

##### Diversity of Medical Specialties

The nurse experts showed a very high level of consensus, despite the diverse background of clinical settings and medical specialties. Some participants mentioned that it would have been interesting to detail the results by type of ward; however, the research team justified aggregating the data because guaranteeing participants’ anonymity required not being able to recognize them from their professional backgrounds.

Breaking data down by type of ward or clinical setting might have had a negative influence on items that did not reach consensus, such as dealing with handovers at the patient's bedside or presenting their medication list.

##### Consensus Reached

The item relating to staff attitudes during handovers—hopefully in a spirit of cooperation—was very favorably received, and had been chosen with regard to the hospital’s declared aims toward collaborative practices, which are part of its strategy and philosophy.

An overview of all the statements accepted by the entire panel was presented to the 15 attending nurse experts. Participants reaffirmed the important role of organizational issues (enough time, suitable staffing levels, appropriate environments, etc) in well-functioning nursing handovers, as shown in the item list. Organizational issues are always present in hospital systems, but health care professionals often perceive them to be obstacles. Designing new nursing handover standards was viewed as an opportunity to align the visions of management and clinicians. Participants also mentioned the limitations and risks relating to changes in practice. The following quote illustrates this:

*Organizational limitations meant that, in general, professionals had not adopted a consensus on how to carry out handovers at the patient's bedside.*

The focus groups offered the opportunity to discuss each statement that had reached a consensus. All the participants, no matter their age, sex, or nursing specialty, were enthusiastic about the consensuses found. The following quotes illustrate their positive mindsets:

I’m happy that these topics found a formal consensus. This will be a great help in formalizing communication between our nurses during shift changes: it will reduce the time spent and hopefully prevent some of the endless disagreements between nurses about which information is pertinent or not...

...Communication on our ward is poorly structured and not always considered as a potential trigger for errors or even conflicts. Standardizing will be a great help...

...this will be an excellent starting point from which to construct our own, adapted, standardized handovers at shift changes on our ward...

##### Consensus Not Reached

Providing a medication list at handover failed to reach the required level of consensus, giving rise to quite heterogeneous opinions among focus group participants. Some stated that it would be difficult to remove this item from standard handover procedures. Others explained that a fraction of the nurse experts replied negatively because a list of medication has little meaning without parameters such as the mode of administration, effects, or follow-up. This is in line with the survey subheading of environmental diversity (ie, different wards and specialties), which mentioned the following:

A more detailed handover may be required, depending on the specialties.

The following was also mentioned:

Perhaps in some wards we don't need to transmit that information verbally since it's in the written part of the file.

Several participants speculated on the different causes of medication errors that are not the result of handover processes:

It would be interesting to see what medication errors are related to, and I don't know if there really is a link, at that time, to the handover. I think there are other problems with medication errors; I don't really think that they are linked to handovers.

I agree. I think that you have to be very strict on procedures—double-checking, not giving medication without consulting the paperwork. I think that errors come from the huge amount of paperwork most of the time.

Suggestions on whether to transmit all medication information were also explored:

It's more focused on clinical problems, problems they have—patients' problems—and nurses make the connections to the drugs.

This degree of detail will be defined in the handover standard’s different sections, including priorities related to time management, mission, and risk management failures:

We clearly want safety, but we cannot afford to make extremely time-consuming reports. Indeed, if we set ourselves a framework at the beginning, and imagine that we have half an hour to do the handover, we will also have to adapt our priorities.

##### Handovers at the Patient's Bedside

Participants’ understanding of the concept of handovers at the patient's bedside may have been different according to their different work settings, and this could have influenced our findings. Handover at the patient's bedside refers to the patient's presence during that handover. The concept also highlighted professionals’ uncertainties regarding potential breaches of confidentiality versus developing a better understanding of patients’ values and preferences during a handover at their bedside. One focus group participant mentioned that her colleagues gave such handovers a lot of thought, stating the following:

...we should try, actually, but there are a lot of questions still to sort out...

##### Information Technology Support

IT support is essential to ensuring the continuity of information transfer. A handover can be summarized using written documentation, allowing professionals guaranteed access to an overview of the data. There was a unanimous consensus on complete data transparency, making it possible to answer any questions that arose during the handover. Currently, however, not all the hospital’s wards have the tools that correspond best to their specialty. Participants agreed that knowing the medication’s precise formulation was a guarantee of safety and continuity. Although there is a recognized risk of errors, paper notes are increasingly used to compensate for the lack of precision or flexibility in electronic patient records.

## Discussion

### Principal Findings

In addition to selecting which items should be included in an evidence-based, shift-to-shift nursing handover standard, this study sought to find a consensus about information flows, best practices, and patient involvement. The significant number of nurse experts involved in our survey determined the need for an electronic data collection method [30,53,54].

The potential benefits arising from this study are due to its combined use of clinical and applied research skills to solve a patient safety issue. Indeed, the study will have a direct impact on future patient safety and the quality and continuity of care in our multisite public hospital in Switzerland. The hospital-wide standard for shift-to-shift nursing handovers will enable frontline nurses in the French-speaking and German-speaking hospital centers to build their own consensus positions on the content necessary for nursing shift handovers and patient transfers within and between different regional care units. Nevertheless, after two rounds of online investigation, some of the nursing professionals would have preferred a single, immediately implementable, nursing handover standard applicable to all the hospital’s care units.

It is also very likely that some item responses were influenced by a reluctance to change, because changing well-established practices could initially induce handover errors. Items that did not reach a hospital-wide consensus could be reconsidered by individual care units or even by hospital centers. Indeed, Flemming and Hübner reported that the inaccurate transmission of medication prescriptions was a frequent type of error [55]. According to the WHO position, more than 40% of prescription and administration errors occur during handovers and transfers—mistakes that could be avoided by medication reconciliation at those moments. This process compares the patient’s list of usual drugs with a new list including deprescriptions and new prescriptions, modified following medical decisions [56]. This item may, therefore, have to be reconsidered at the French-speaking hospital center in the future—but not at the German-speaking hospital center—as may the nurse experts’ feelings about bedside handovers, which allow patients to express their decisions, opinions, and expectations, and validate their latest clinical data and medication. This is especially relevant, since the French-speaking hospital center’s nurse experts did not perceive potential breaches of confidentiality during bedside handovers to be a risk. About two-thirds of the French-speaking hospital center’s nurse experts were not convinced that this item needed to be included in the nursing handover standard; however, the German-speaking hospital center’s nurse experts were interested in adopting it. These contrasting results might be explained by differences in the proportions of managers and full-time employees at the two centers, or by differences in understanding or training about the concept of bedside handovers. Use of a mnemonic tool did not achieve consensus at the German-speaking hospital center, despite mental models in the form of acronyms being encouraged as a strategy to improve handover process safety, especially in situations where large amounts of sometimes disparate information are communicated [19]. Despite the differences in the second-round items resubmitted to participants at the two hospital centers, the findings presented here will be a significant contribution to our multisite public hospital’s overall strategy of seeking continual improvements in the quality and safety of care via evidence-based practices [57]. As Klee et al and McFarlane stated, creating a consensus handover standard is a way to change the daily practices of all the nurses involved in this nursing process, and its results are not limited to those who accepted the item statements [58,59]. This e-Delphi survey process should not be considered as a generalizable method for creating clinical nursing handover standards in other hospitals. However, the approach could be a starting point for developing good clinical research practice in large samples. The final consensus position will be decisive in implementing our hospital’s new nursing handover strategy at ward level. Encouraging a high survey participation rate will contribute to motivating nursing teams to really change their practice. Nursing management teams should nevertheless reflect on how to support and supervise nursing professionals as they attempt to adapt items to the specificities of their own care units.

### Strengths and Limitations

This study’s greatest strength was its high participation rate from among the potential sample of experts. There was a positive response to the survey because it addressed a theme of concern to nursing teams’ daily practice and its results might benefit them directly and rapidly. Another explanation could be that developing a participative consensus, giving experts the opportunity to express themselves and submit proposals concerning their working environment, meant that their expertise was recognized by the management of their multisite public hospital. The work’s added value probably lies in its scientific rigor, particularly questionnaire development using an evidence-based scoping review. Giving clinical experts, who are active in so many disciplines, the opportunity to critically analyze the standard may have contributed to the high level of consensus reached. This high level of consensus, communicated to the participants during the focus group presentation, made the methodology clear to everyone. The reflection period before the adoption of the handover standard by the different care units could be considered a strength and a limitation.

Our study’s first limitation concerns the probability that some item results were influenced by a reluctance to change, thus inducing more positive or negative responses, depending on the item. A second limitation was that all the hospital’s clinical specialties were involved, making it likely that the consensus was biased toward those specialties represented by the greatest numbers of nurse experts. We did not analyze the collected data by medical specialty in order to ensure participant anonymity. Information is also more difficult to coordinate across a multisite hospital with centers relatively distant from one another. It should also be noted that an online survey makes it impossible to ensure that participants gave their responses autonomously and without peer influence. Another limitation concerned the discrepancy in round 2’s e-Delphi items—resulting from answers to the open-ended question—which were not all resubmitted to the two different hospital centers.

A more organizational limitation to constructing standardized handovers is that there is no guarantee of its implementation and optimal use. Nurses will have to be trained on how to use a standardized handover tool, with a tailored implementation strategy for each ward and department. Future research should examine the effectiveness of the standardized handover’s introduction, using quasi-experimental intervention studies (ie, before and after), completed with postimplementation satisfaction surveys: qualitative surveys among nurses (ie, focus groups) and quantitative surveys among patients (ie, online surveys and questionnaires at the end of hospitalization). Finally, error rates (eg, medication, clinical follow-up of unstable patients, and so on) before and after the implementation should also be compared.

### Conclusions

A standardized, hospital-wide, shift-to-shift nursing handover process encourages nursing care teams to conscientiously share information that is essential to the continuity of care. This participative study enabled us to reveal a high level of consensus on a large majority of the items proposed for such a nursing handover standard. Effective compliance with the new standard will be the expression of its successful implementation. However, further dimensions of nursing handovers have yet to be explored, particularly on the causes of the risks of error and on the interprofessional sharing of information that enables the coordination of patient-centered care. Proactive leadership from hospital management and appropriate staff training will be the next crucial steps toward the successful implementation of our institution-wide standard for evidence-based nursing handovers between shifts and care units.

## Abbreviations

CER-VD: Human Research Ethics Committee of the Canton Vaud
CINAHL: Cumulative Index to Nursing and Allied Health Literature
e-Delphi: electronic Delphi
HES-SO: Haute École Spécialisée Suisse orientale
IT: information technology
MEDLINE: Medical Literature Analysis and Retrieval System Online
WHO: World Health Organization

## References

[1] Blouin AS (2011). Improving hand-off communications. J Nurs Care Qual.

[2] Jeffs L, Acott A, Simpson E, Campbell H, Irwin T, Lo J, Beswick S, Cardoso R (2013). The value of bedside shift reporting enhancing nurse surveillance, accountability, and patient safety. J Nurs Care Qual.

[3] Jeffs L, Beswick S, Acott A, Simpson E, Cardoso R, Campbell H, Irwin T (2014). Patientsʼ views on bedside nursing handover. J Nurs Care Qual.

[4] The Australian Resource Centre for Healthcare Innovations (2005). Clinical Handover and Patient Safety Literature Review Report.

[5] Catchpole K, Sellers R, Goldman A, McCulloch P, Hignett S (2010). Patient handovers within the hospital: Translating knowledge from motor racing to healthcare. Qual Saf Health Care.

[6] Matthaeus-Kraemer CT, Thomas-Rueddel DO, Schwarzkopf D, Rueddel H, Poidinger B, Reinhart K, Bloos F (2016). Crossing the handover chasm: Clinicians' perceptions of barriers to the early detection and timely management of severe sepsis and septic shock. J Crit Care.

[7] Hübner U, Przysucha M (2017). Patient handovers - cognitively demanding: Does the handover EHR meet this challenge?. Stud Health Technol Inform.

[8] Manser T, Foster S (2011). Effective handover communication: An overview of research and improvement efforts. Best Pract Res Clin Anaesthesiol.

[9] WHO Collaborating Centre for Patient Safety Solutions (2007). Communication During Patient Hand-Overs.

[10] Slade D, Murray KA, Pun JK, Eggins S (2019). Nurses' perceptions of mandatory bedside clinical handovers: An Australian hospital study. J Nurs Manag.

[11] Pun J, Chan EA, Eggins S, Slade D (2020). Training in communication and interaction during shift-to-shift nursing handovers in a bilingual hospital: A case study. Nurse Educ Today.

[12] VanGraafeiland B, Foronda C, Vanderwagen S, Allan L, Bernier M, Fishe J, Hunt EA, Jeffers JM (2019). Improving the handover and transport of critically ill pediatric patients. J Clin Nurs.

[13] Spurgeon P, Sujan M, Cross S, Flanagan H (2019). A systems approach to improving clinical handover in emergency care. Building Safer Healthcare Systems: A Proactive, Risk Based Approach to Improving Patient Safety.

[14] Spooner AJ, Chaboyer W, Aitken LM (2019). Interruptions during senior nurse handover in the intensive care unit. J Nurs Care Qual.

[15] Sanjuan-Quiles Á, Hernández-Ramón MD, Juliá-Sanchis R, García-Aracil N, Castejón-de la Encina ME, Perpiñá-Galvañ J (2019). Handover of patients from prehospital emergency services to emergency departments. J Nurs Care Qual.

[16] Malfait S, Eeckloo K, Van Biesen W, Van Hecke A (2019). Barriers and facilitators for the use of nursing bedside handovers: Implications for evidence-based practice. Worldviews Evid Based Nurs.

[17] Bukoh MX, Siah CR (2020). A systematic review on the structured handover interventions between nurses in improving patient safety outcomes. J Nurs Manag.

[18] Welsh CA, Flanagan ME, Ebright P (2010). Barriers and facilitators to nursing handoffs: Recommendations for redesign. Nurs Outlook.

[19] The Joint Commission (2017). New alert focuses on safety culture in health care. Jt Comm Perspect.

[20] (2017). Rapport Qualité 2017: Résumé Hôpital du Valais.

[21] Ferguson TD, Howell TL (2015). Bedside reporting: Protocols for improving patient care. Nurs Clin North Am.

[22] Fagerlund A, Keebler JR, Lew V, Lazzara EH, Welsh K (2016). Team performance framework during handoffs. Proceedings of the Human Factors and Ergonomics Society Annual Meeting.

[23] Benson E, Rippin-Sisler C, Jabusch K, Keast S (2007). Improving nursing shift-to-shift report. J Nurs Care Qual.

[24] Toakley C, Green C (2016). ISBAR-ICU: Development and implementation of a standardised ICU clinical handover tool: Nursing perceptions, barriers and challenges. Proceedings of the 12th Congress of the World Federation of Critical Care Nurses (WFCCN).

[25] Holly C, Poletick EB (2014). A systematic review on the transfer of information during nurse transitions in care. J Clin Nurs.

[26] Ferorelli D, Giandola TP, Laterza M, Solarino B, Pezzolla A, Zotti F, Dell'Erba A (2017). Handover checklist: Testing a standardization process in an Italian hospital. Risk Manag Healthc Policy.

[27] The Joint Commission (2017). Inadequate hand-off communication. Sentinel Event Alert.

[28] Keebler JR, Lazzara EH, Patzer BS, Palmer EM, Plummer JP, Smith DC, Lew V, Fouquet S, Chan YR, Riss R (2016). Meta-analyses of the effects of standardized handoff protocols on patient, provider, and organizational outcomes. Hum Factors.

[29] Jukkala AM, James D, Autrey P, Azuero A, Miltner R (2012). Developing a standardized tool to improve nurse communication during shift report. J Nurs Care Qual.

[30] Keeney S, Hasson F, McKenna H (2011). The Delphi Technique in Nursing and Health Research.

[31] Cole ZD, Donohoe HM, Stellefson ML (2013). Internet-based Delphi research: Case based discussion. Environ Manage.

[32] Jünger S, Payne SA, Brine J, Radbruch L, Brearley SG (2017). Guidance on Conducting and REporting DElphi Studies (CREDES) in palliative care: Recommendations based on a methodological systematic review. Palliat Med.

[33] Tong A, Sainsbury P, Craig J (2007). Consolidated criteria for reporting qualitative research (COREQ): A 32-item checklist for interviews and focus groups. Int J Qual Health Care.

[34] World Medical Association (1997). World Medical Association Declaration of Helsinki. Recommendations guiding physicians in biomedical research involving human subjects. JAMA.

[35] Jayasekara RS (2012). Focus groups in nursing research: Methodological perspectives. Nurs Outlook.

[36] Graan SM, Botti M, Wood B, Redley B (2016). Nursing handover from ICU to cardiac ward: Standardised tools to reduce safety risks. Aust Crit Care.

[37] Smeulers M, Lucas C, Vermeulen H (2014). Effectiveness of different nursing handover styles for ensuring continuity of information in hospitalised patients. Cochrane Database Syst Rev.

[38] Hochman BR, Barry ME, Lane-Fall MB, Allen SR, Holena DN, Smith BP, Kaplan LJ, Pascual JL (2017). Handoffs in the intensive care unit. Am J Med Qual.

[39] Walsh J, Messmer PR, Hetzler K, O'Brien DJ, Winningham BA (2018). Standardizing the bedside report to promote nurse accountability and work effectiveness. J Contin Educ Nurs.

[40] Eltaybani S, Mohamed N, Abdelwareth M (2019). Nature of nursing errors and their contributing factors in intensive care units. Nurs Crit Care.

[41] Zou X, Zhang Y (2016). Rates of nursing errors and handoffs-related errors in a medical unit following implementation of a standardized nursing handoff form. J Nurs Care Qual.

[42] Tacchini-Jacquier N, de Waele E, Urben P, Turini P, Verloo H (2020). Developing an evidence-based nursing handover standard for a multi-site public hospital in Switzerland: Protocol for a web-based, modified Delphi study. JMIR Res Protoc.

[43] Burchell AN, Lisk R, Yeung A, Rana J, Bacon J, Brunetta J, Gilbert M, Gesink D, Grewal R, Guiang CB, Kwag M, Logie CH, Mitterni L, Shahin R, Tan DH (2019). Community-directed bacterial sexually transmitted infection testing interventions among men who have sex with men: Protocol for an e-Delphi study in Toronto, Canada. JMIR Res Protoc.

[44] Slade SC, Dionne CE, Underwood M, Buchbinder R, Beck B, Bennell K, Brosseau L, Costa L, Cramp F, Cup E, Feehan L, Ferreira M, Forbes S, Glasziou P, Habets B, Harris S, Hay-Smith J, Hillier S, Hinman R, Holland A, Hondras M, Kelly G, Kent P, Lauret G, Long A, Maher C, Morso L, Osteras N, Peterson T, Quinlivan R, Rees K, Regnaux J, Rietberg M, Saunders D, Skoetz N, Sogaard K, Takken T, van Tulder M, Voet N, Ward L, White C (2016). Consensus on Exercise Reporting Template (CERT): Modified Delphi study. Phys Ther.

[45] World Medical Association (1996). Declaration of Helsinki (1964): Recommendations guiding physicians in biomedical research involving human subjects. BMJ.

[46] Patrick DL, Burke LB, Gwaltney CJ, Leidy NK, Martin ML, Molsen E, Ring L (2011). Content validity--Establishing and reporting the evidence in newly developed patient-reported outcomes (PRO) instruments for medical product evaluation: ISPOR PRO good research practices task force report: Part 1--Eliciting concepts for a new PRO instrument. Value Health.

[47] Jayasekara RS (2012). Focus groups in nursing research: Methodological perspectives. Nurs Outlook.

[48] IBM Corporation (2019). IBM.

[49] Elo S, Kyngäs H (2008). The qualitative content analysis process. J Adv Nurs.

[50] Hsieh H, Shannon SE (2005). Three approaches to qualitative content analysis. Qual Health Res.

[51] (2020). QSR International.

[52] (2020). Swiss Health Promotion.

[53] de Souza BR, Avelar MC (2009). Nursing diagnosis in care of patients with renal insufficiency acute: Delphi technique. Online Braz J Nurs.

[54] O'Rourke J, Abraham J, Riesenberg LA, Matson J, Lopez KD (2018). A Delphi study to identify the core components of nurse to nurse handoff. J Adv Nurs.

[55] Flemming D, Hübner U (2013). How to improve change of shift handovers and collaborative grounding and what role does the electronic patient record system play? Results of a systematic literature review. Int J Med Inform.

[56] World Health Organization (2016). Transitions of Care: Technical Series on Safer Primary Care.

[57] Boulkedid R, Abdoul H, Loustau M, Sibony O, Alberti C (2011). Using and reporting the Delphi method for selecting healthcare quality indicators: A systematic review. PLoS One.

[58] Klee K, Latta L, Davis-Kirsch S, Pecchia M (2012). Using continuous process improvement methodology to standardize nursing handoff communication. J Pediatr Nurs.

[59] McFarlane A (2018). The impact of standardised perioperative handover protocols. J Perioper Pract.

